# Cross-Modal Transformer-Based Streaming Dense Video Captioning with Neural ODE Temporal Localization

**DOI:** 10.3390/s25030707

**Published:** 2025-01-24

**Authors:** Shakhnoza Muksimova, Sabina Umirzakova, Murodjon Sultanov, Young Im Cho

**Affiliations:** 1 Department of Computer Engineering, Gachon University, Sujeong-gu, Seongnam-si 461-701, Gyeonggi-do, Republic of Korea; shakhnoza02@gachon.ac.kr; 2 Department of Information Systems and Technologies of the Tashkent State University of Economic, Tashkent 100066, Uzbekistan; murad.sultanov@tsue.uz

**Keywords:** cross-modal transformer, streaming dense video captioning, neural ODE temporal localization, cross-modal memory retrieval, multi-scale transformer decoder, real-time processing

## Abstract

Dense video captioning is a critical task in video understanding, requiring precise temporal localization of events and the generation of detailed, contextually rich descriptions. However, the current state-of-the-art (SOTA) models face significant challenges in event boundary detection, contextual understanding, and real-time processing, limiting their applicability to complex, multi-event videos. In this paper, we introduce CMSTR-ODE, a novel Cross-Modal Streaming Transformer with Neural ODE Temporal Localization framework for dense video captioning. Our model incorporates three key innovations: (1) Neural ODE-based Temporal Localization for continuous and efficient event boundary prediction, improving the accuracy of temporal segmentation; (2) cross-modal memory retrieval, which enriches video features with external textual knowledge, enabling more context-aware and descriptive captioning; and (3) a Streaming Multi-Scale Transformer Decoder that generates captions in real time, handling objects and events of varying scales. We evaluate CMSTR-ODE on two benchmark datasets, YouCook2, Flickr30k, and ActivityNet Captions, where it achieves SOTA performance, significantly outperforming existing models in terms of CIDEr, BLEU-4, and ROUGE scores. Our model also demonstrates superior computational efficiency, processing videos at 15 frames per second, making it suitable for real-time applications such as video surveillance and live video captioning. Ablation studies highlight the contributions of each component, confirming the effectiveness of our approach. By addressing the limitations of current methods, CMSTR-ODE sets a new benchmark for dense video captioning, offering a robust and scalable solution for both real-time and long-form video understanding tasks.

## 1. Introduction

Dense video captioning is a crucial task in video understanding, requiring the localization of multiple events in long, untrimmed videos and the generation of detailed natural language descriptions for each event [1,2]. This capability is essential for applications such as video surveillance, content summarization, autonomous systems, and accessibility solutions for visually impaired users. However, dense video captioning presents significant challenges due to the complexity of event detection [3], the need for real-time caption generation [4], and the variety of visual information that must be processed.

Current SOTA models for dense video captioning, such as OW-VISCap [5], OmniViD [6], and TrafficVLM [7], have made significant advancements by incorporating multi-modal features, hierarchical event localization, and transformer-based architectures. These innovations have allowed the models to improve their ability to handle complex video data and generate more accurate captions. However, despite these advancements, these models continue to face several critical limitations. First, temporal localization, or the precise identification of the start and end of events, remains a significant challenge. This is particularly evident in long, untrimmed videos [8], where multiple events may overlap or occur in rapid succession, making it difficult for the models to segment events accurately. Second, many of these models struggle with contextual understanding. They often rely heavily on visual features, which limits their capacity to generate contextually rich and descriptive captions. In cases where the visual cues are insufficient or ambiguous, these models are unable to incorporate additional context, resulting in captions that lack depth and are less informative. Finally, real-time performance is a persistent challenge for existing models. Many of the current approaches process videos in a batch mode, meaning they require the entire video to be available before they can generate captions. This processing method restricts their use in real-time scenarios, such as live video feeds, where captions need to be generated on the fly as the video is being played. Addressing these challenges is crucial for advancing dense video captioning models, particularly for applications that require accurate event segmentation, enriched contextual descriptions, and real-time performance.

To address the limitations of current video captioning systems, we introduce CMSTR-ODE, a novel framework that integrates a Cross-Modal Streaming Transformer with Neural ODE Temporal Localization. This model significantly enhances event localization, contextual understanding, and real-time performance. The Neural ODE-based Temporal Localization efficiently models the dynamics of video frames, ensuring precise and continuous event detection even in scenarios with overlapping or rapidly occurring events. This improves the model ability to handle complex videos and boosts accuracy in temporal localization. Additionally, the cross-modal memory retrieval feature enriches the captioning process by integrating external textual information, enabling the generation of richer, more contextually relevant captions when visual cues are ambiguous. Finally, the Streaming Multi-Scale Transformer Decoder processes frames as they arrive, allowing for real-time, incremental caption generation. Its multi-scale attention mechanism ensures that all details, regardless of object size, are captured accurately, making CMSTR-ODE ideal for live video applications where captions must be generated on the fly without compromising quality. These innovations enable CMSTR-ODE to address the core challenges of dense video captioning. First, our model achieves precise event localization through the use of Neural Ordinary Differential Equations (ODEs), which model the continuous evolution of video features over time, resulting in superior temporal segmentation. Second, by leveraging cross-modal memory retrieval, our model retrieves relevant semantic information from external textual sources, enriching the generated captions with contextual depth. Finally, the streaming decoder allows for real-time caption generation, making CMSTR-ODE suitable for time-sensitive applications.

Our contributions are summarized as follows:

- We introduce a Neural ODE-based Temporal Localization module that improves event boundary detection accuracy and reduces the computational overhead associated with conventional recurrent neural networks (RNNs) or transformers.
- We propose a cross-modal memory retrieval mechanism to augment visual features with text-based contextual knowledge, enhancing the richness and relevance of the generated captions.
- We develop a Streaming Multi-Scale Transformer Decoder that enables real-time caption generation while maintaining high accuracy across multiple scales of visual features.

We evaluate CMSTR-ODE on two benchmark datasets, YouCook2, Flickr30k, and on ActivityNet Captions, and demonstrate that our model outperforms SOTA methods across several metrics, including CIDEr, BLEU-4, and ROUGE. Additionally, we provide a detailed ablation study to highlight the contributions of each component in our framework. The rest of the paper is organized as follows: Section 2 discusses related work, highlighting the progress and limitations of current dense video captioning models. Section 3 introduces the architecture and components of CMSTR-ODE in detail. Section 4 presents the experimental results, including performance comparisons with SOTA models; Section 5 provides a discussion; and Section 6 concludes with a discussion of the model strengths and future directions.

## 2. Literature Review

Dense video captioning, a complex task involving temporal localization of events and the generation of natural language descriptions, has seen rapid advancements in recent years [9,10]. Several models have been proposed, each targeting different aspects of this challenge, such as event boundary detection [11,12], multi-modal learning [13,14], and real-time processing. In this section, we review the key contributions in video captioning, focusing on recent developments in temporal event localization, multi-modal video understanding, and streaming video captioning. Additionally, we discuss the limitations of the current SOTA models and how our CMSTR-ODE addresses these challenges.

### 2.1. Temporal Event Localization in Video Captioning

Accurately localizing events within long, untrimmed videos is a core challenge in dense video captioning. Early works focused on proposal-based methods, where candidate segments were generated and ranked based on their relevance to the event descriptions. For example, Vid2Seq [15] uses time tokens to generate event boundaries and textual descriptions in a unified sequence, achieving improvements in temporal precision. Similarly, ACNet [16] introduces auxiliary captions for video grounding, which helps mitigate the sparsity of annotated data and enhances the localization of events by using additional contextual information. However, these proposal-based methods often struggle with overlapping or closely occurring events, as they rely on pre-defined segment proposals that may not align with the actual event boundaries. In contrast, recent proposal-free methods, such as those used in OmniViD [6] and TrafficVLM [7], directly predict event boundaries without generating candidate proposals. While effective in some scenarios, these methods still face challenges in accurately modeling the temporal dynamics of complex, multi-event videos.

To address these limitations, CMSTR-ODE introduces a Neural ODE-based Temporal Localization mechanism. This approach models the continuous evolution of video features over time using Neural ODEs, enabling precise event boundary detection while reducing the computational cost associated with traditional recurrent models. By treating the temporal aspect of videos as a continuous process, CMSTR-ODE improves localization accuracy, especially in scenarios involving overlapping or subtle events.

### 2.2. Multi-Modal Learning for Video Captioning

Dense video captioning requires not only accurate event localization but also the generation of rich, context-aware descriptions. To achieve this, models must integrate information from multiple modalities, such as visual, auditory, and textual data. Early works in multi-modal learning primarily focused on video–text alignment, where video features were mapped to a common embedding space with text representations. For instance, Video ReCap [17] adopts a hierarchical video-captioning approach, leveraging clip-level and segment-level descriptions to generate captions for long videos. More recent approaches, such as TrafficVLM [7] and ACNet [16], incorporate dense video captioning models with cross-modal attention mechanisms, which allow the models to learn joint representations from both video and textual data. This helps improve the quality of the generated captions, especially in scenarios where the visual features alone are insufficient for comprehensive descriptions.

However, existing multi-modal approaches often lack mechanisms to incorporate external contextual knowledge beyond the immediate video content. CMSTR-ODE overcomes this limitation with its cross-modal memory retrieval mechanism. This module retrieves relevant textual information from an external memory bank, enriching the visual features with semantic context derived from related text embeddings. This allows the model to generate more detailed and contextually accurate captions, even when the video lacks explicit visual cues for certain events.

### 2.3. Streaming Video Captioning

With the increasing need for real-time applications, such as live video captioning and video surveillance, the demand for efficient streaming models has grown [18]. Traditional video captioning models typically process the entire video sequence before generating captions, making them unsuitable for real-time use [19]. To address this, recent models have begun incorporating streaming mechanisms that enable incremental caption generation. For example, OmniViD [6] uses a unified encoder–decoder framework that can handle various video tasks, including dense video captioning, through a single model architecture. However, while OmniViD supports multiple tasks, it still processes videos in fixed-length sequences, limiting its real-time applicability. Similarly, TrafficVLM [7] incorporates a multi-phase description mechanism that allows it to generate long descriptions for traffic events in real time, but its performance is optimized for domain-specific tasks, limiting its generalizability to other video types.

To address these challenges, CMSTR-ODE introduces a Streaming Multi-Scale Transformer Decoder that processes video frames as they arrive, generating captions in real time without sacrificing accuracy. This streaming approach allows the model to handle long videos incrementally, making it suitable for time-sensitive applications. Additionally, the multi-scale attention mechanism enables the model to capture objects and events of varying sizes, improving the precision of captions across different spatial scales.

### 2.4. Recursive and Hierarchical Video Captioning

Long-range video understanding requires models that can capture both low-level atomic actions and high-level goals, especially in scenarios where videos have hierarchical structures, such as human activities in egocentric videos. Video ReCap [17] introduces a recursive video-captioning architecture that generates captions at multiple levels of hierarchy, from short clip captions to long-range video summaries. The model uses curriculum learning to gradually learn hierarchical structures, enabling it to generate detailed captions for both short and long videos. While hierarchical approaches like Video ReCap are effective for long-form videos, they often struggle with real-time caption generation and can be computationally intensive due to the recursive nature of the architecture. CMSTR-ODE, by contrast, combines real-time streaming capabilities with the ability to capture multi-scale visual features, making it more efficient for both short and long videos.

### 2.5. Limitations of SOTA Models

While the current SOTA models have achieved notable progress in video captioning, several key limitations remain. One significant issue is event localization. Models such as OmniViD [6] and ACNet [16] often struggle with accurately detecting event boundaries, particularly in complex videos where multiple events overlap or happen in rapid succession. This challenge limits their ability to segment videos with precision, which is crucial for generating accurate captions. Another major limitation is real-time processing. Models like Video ReCap [17] and OW-VISCap [5] perform well on long, pre-recorded videos, but they are not optimized for real-time streaming applications. Their inability to generate captions on the fly makes them less suitable for scenarios such as live video feeds or time-sensitive applications where immediate captioning is essential. Finally, contextual understanding poses a challenge for most existing models. These models tend to rely heavily on visual features without incorporating external knowledge, making it difficult for them to produce rich, descriptive captions in ambiguous or complex scenes. The lack of mechanisms to integrate additional contextual information limits their ability to interpret nuanced events or generate more detailed and meaningful descriptions. Addressing these challenges is critical for advancing the performance of video captioning models, particularly in real-time and complex video environments.

CMSTR-ODE addresses these challenges by introducing a more precise temporal localization mechanism (Neural ODE-based), an external memory retrieval system for enriched context, and a real-time streaming decoder capable of handling multi-scale visual features. While existing SOTA models have achieved impressive results in dense video captioning, they face challenges in event localization accuracy, real-time processing, and contextual understanding. Our proposed CMSTR-ODE framework builds upon these advances, incorporating novel techniques like Neural ODE-based Temporal Localization, cross-modal memory retrieval, and a Streaming Multi-Scale Transformer Decoder to push the boundaries of dense video captioning performance. By addressing the limitations of previous models, CMSTR-ODE achieves superior performance across multiple benchmarks, setting a new standard for both real-time and long-form video captioning tasks.

## 3. Proposed Method: CMSTR-ODE

This section introduces the CMSTR-ODE framework, a novel solution for dense video captioning that overcomes key challenges such as precise event localization, contextual understanding, and real-time caption generation. Our approach integrates advanced techniques to ensure accurate temporal segmentation and rich, descriptive captions while maintaining computational efficiency for real-time applications. The framework employs Neural ODE-based Temporal Localization to model the continuous evolution of video features, allowing for the precise detection of event boundaries even in complex, overlapping scenarios. A cross-modal memory retrieval mechanism is incorporated to enhance the model contextual understanding, enriching video features with relevant external textual knowledge. This ensures that the captions generated are accurate, contextually rich, and meaningful. Finally, the Streaming Multi-Scale Transformer Decoder processes video frames incrementally, enabling real-time caption generation. By attending to objects and events at multiple scales, the decoder captures both fine-grained details and broader visual contexts, resulting in more comprehensive descriptions. This section provides a detailed explanation of the architecture, processes, and underlying algorithms that make up the CMSTR-ODE framework, highlighting its ability to address the limitations of the current state-of-the-art methods.

**Figure 1:**
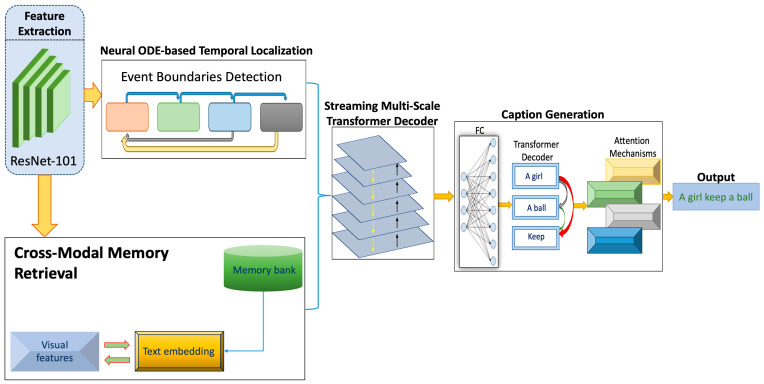
Architecture of a cross-modal neural network for event detection and captioning.

Figure 1 depicts an integrated architecture for video processing and caption generation. The process initiates with feature extraction using ResNet-101, followed by event boundary detection via a Neural ODE-based temporal localization. The architecture incorporates a cross-modal memory retrieval system that aligns visual features with text embeddings, enhancing the system contextual understanding. A Streaming Multi-Scale Transformer Decoder addresses temporal variations in the data effectively. The caption generation module, utilizing a transformer decoder and attention mechanisms, generates accurate and context-sensitive captions, such as ‘A girl keeps a ball’, illustrating the system capability to synthesize visual and textual data into coherent outputs.

Algorithm 1 outlines the CMSTR-ODE framework for dense video captioning, integrating temporal event localization, cross-modal knowledge retrieval, and streaming caption generation. This algorithm processes an untrimmed video sequence in real time to detect events and generate accurate captions. It initializes with three key components: visual features, event boundaries, and output captions. During the feature extraction phase, it extracts visual features using a pre-trained encoder like a CNN or transformer. These features form the basis for event detection and caption generation. In the Neural ODE-based Temporal Localization step, the algorithm uses Neural ODEs to model the temporal dynamics of video frames, predicting precise event boundaries even in scenarios with overlapping events. Following event localization, the cross-modal memory retrieval phase enriches visual features with relevant textual knowledge from an external memory bank, ensuring the captions reflect the broader contextual information. The final phase, the Streaming Multi-Scale Transformer Decoder, processes these enriched features to generate captions in real time. Its multi-scale attention mechanism adjusts focus across different object sizes within the video, capturing detailed descriptions. Captions are produced incrementally as frames are processed, facilitating real-time performance without needing the full video. This integration of Neural ODE-based segmentation, cross-modal memory, and streaming decoding positions the CMSTR-ODE framework as ideal for real-time dense video captioning applications. To maintain an optimal balance between memory usage and update speed, we implement an active memory pruning mechanism. This system regularly evaluates the relevance and frequency of use of stored data, removing outdated or infrequently accessed information. This dynamic pruning helps keep the memory size manageable and the retrieval process efficient.
**Algorithm 1**. CMSTR-ODE for Dense Video Captioning**Input**: Untrimmed video sequence *V* = {*F*_1_, *F*_2_,…, *F_T_*} where *F_t_* represents each video frame **Output**: Captions *C* = {*C*_1_, *C*_2_,…, *C_N_*} for detected events
1. **Initialize**: - Feature set: VisualFeatures = [] - Event boundaries: EventBoundaries = [] - Output captions: C = []
2. **Step 1**: Feature Extraction
3. for each frame *F_t_* in video *V*
**do**
4.   *v_t_* = ExtractFeatures(*F_t_*) //Extract visual features using CNN/Transformer
5.   **Append** *v_t_* to VisualFeatures//Store visual features for further processing
6. **end for**
7. **Step 2**: Neural ODE-based Temporal Localization
8. for each frame feature *v_t_* in VisualFeatures **do**
9.   *start_t_*, *end_t_* = NeuralODE(*v_t_*)//Predict start and end boundaries using Neural ODE
10.  **if**
*start_t_* or *end_t_* detected **then** 11.      Append (*start_t_*, *end_t_*) to EventBoundaries//Store detected event boundaries
12.  **end if**
13. **end for**
14. **Step 3**: Cross-Modal Memory Retrieval
15. **for each** event segment (*start_t_*, *end_t_*) in EventBoundaries **do**
16.   *H* = []//Initialize list for enriched features
17.   **for each** frame feature *v_t_* in segment (*start_t_*, *end_t_*) do
18.    *e_t_* = RetrieveFromMemory(*v_t_*)//Retrieve text embedding from memory bank
19.    *h_t_* = *α* × *v_t_* + (1 − *α*) × *e_t_*//Combine visual and text features
20.    Append *h_t_* to *H*//Store enriched features
21.   **end for**
22. **end for**
23. **Step 4**: Streaming Multi-Scale Transformer Decoder
24. **for each** event segment *H* do
25.   *Z* = []//Initialize list for multi-scale attended features
26.   **for each** enriched feature *h_t_* in *H* do
27.    *z_t_* = MultiScaleAttention(*h_t_*)//Apply multi-scale attention mechanism
28.    Append *z_t_* to *Z*//Store the attended features
29.   **end for**
30. **end for**
31. **Step 5**: Caption Generation
32. **for each** event segment *Z* do
33.   *C_i_* = []//Initialize list for caption words
34.   **for each** feature *z_t_* in *Z* do
35.    word = Decoder(*z_t_*)//Generate caption words using streaming decoder
36.    Append word to *C_i_*//Construct caption incrementally
37.   **end for**
38.   Append *C_i_* to *C*//Store the generated caption for the event
39. **end for**
40. **Step 6**: Output Real-Time Captions
41. **return** *C*//Output the generated captions for all detected events

### 3.1. Neural ODE Temporal Localization

Temporal localization is one of the most challenging aspects of dense video captioning. The task involves identifying when specific events occur within a long, untrimmed video and marking their start and end times. Conventional models often rely on recurrent neural networks (RNNs) or long short-term memory (LSTM) architectures to model temporal dependencies across video frames. However, these methods are computationally expensive and struggle with long-range dependencies, especially in the context of dense video captioning, where multiple events may overlap or occur in quick succession. Our proposed solution is to leverage Neural ODEs for efficient and accurate event localization. Neural ODEs provide a continuous and flexible way to model temporal dynamics, enabling us to predict event boundaries more effectively without the computational overhead associated with traditional RNN-based approaches. By modeling the evolution of video features over time as a continuous function, the ODE-based module can capture fine-grained temporal patterns with fewer parameters, making it ideal for real-time and large-scale applications. The primary goal of the Neural ODE Temporal Localization module is to identify precise event boundaries in a video by predicting the start and end times of each event. Unlike conventional methods that use frame-by-frame analysis, our approach models the temporal evolution of video features continuously. This is achieved by solving the ODE governing the temporal changes in video features between frames.

#### Temporal Dynamics of Video Features

Given an input video V consisting of a sequence of frames {$F_{1},$ $F_{2},\ldots$ $F_{T}$}, we extract visual features $x\left(t\right)$ from each frame $F_{t}$ using a pre-trained visual encoder. These features $x\left(t\right)$ are then used as the initial conditions for the Neural ODE:

(1) $$\frac{dx(t)}{dt}=f(x\left(t\right),\;t,\;\theta )$$

where $x\left(t\right)$ represents the extracted visual features at time t, f is a neural network that captures the temporal dynamics of these features, and *θ* denotes the parameters optimized to capture the temporal relationships within the data, facilitating accurate and efficient event localization. The ODE solver integrates this function over time to produce feature representations for subsequent frames. This continuous feature representation allows the model to capture subtle changes in the video, such as the transition between events or the progression of actions within an event. To localize events, we need to predict when these transitions occur. We frame the event localization problem as one of boundary detection, where we aim to predict the start and end times of each event. Using the output of the ODE solver, we apply a boundary detection network that classifies whether a given time step corresponds to the start or end of an event. Formally, for each time step t, the boundary detection network takes the ODE-generated feature $x\left(t\right)$ and outputs a probability score $P_{s}\left(t\right)\;\mathrm{and}\;P_{e}(t)$ representing the likelihood that *t* corresponds to the start or end of an event:

(2) $$P_{start}\left(t\right)=\sigma \left(W_{s}\cdot x\left(t\right)+b_{s}\right),\;P_{end}\left(t\right)=\sigma \left(W_{e}\cdot x\left(t\right)+b_{e}\right)$$

where $W_{s}$, $W_{e}$ and $b_{s}$, $b_{e}$ are learnable parameters and σ is the sigmoid function that maps the output to a probability. During training, we use ground-truth event boundaries to supervise the learning process. The loss function is a combination of binary cross-entropy losses for both the start and end predictions:

(3) $$L_{boundary}=\sum_{t}[BCE(P_{start}\left(t\right),\;Y_{start}(t))+BCE(P_{end}(t),\;Y_{end}(t))]$$

where $Y_{start}(t)$ and $Y_{end}(t)$ are the ground truth labels for the start and end of events at time t, and BCE is the binary cross-entropy loss. One of the key strengths of the Neural ODE-based approach is its ability to handle overlapping events and complex temporal patterns. By modeling the continuous evolution of video features, the ODE-based temporal localization module is able to capture fine-grained transitions between events, even when they overlap or occur in quick succession. This is particularly important in real-world scenarios, where multiple activities may occur simultaneously or where event boundaries are not clearly defined. To handle such cases, we apply a multi-label event detection strategy, where each time step can be associated with multiple event boundaries. The model is trained to predict multiple start and end times for overlapping events, allowing it to localize complex event sequences with high accuracy. A critical aspect of Neural ODEs is the use of numerical solvers to integrate the ODE over time. During inference, we use adaptive step-size solvers that dynamically adjust the number of steps based on the complexity of the video. For simple videos with fewer events, the solver takes larger steps, reducing the computational load. For more complex videos with rapid transitions, the solver takes smaller steps to capture the intricate temporal dynamics. This adaptive inference strategy ensures that the model remains computationally efficient even when processing long videos, while still maintaining high localization accuracy. Our Neural ODE Temporal Localization module provides an efficient and accurate way to localize events in long, untrimmed videos. By modeling the continuous evolution of video features, the ODE-based approach offers significant improvements in temporal localization accuracy while reducing the computational load. Combined with cross-modal memory retrieval and streaming multi-scale decoding, this module forms a core component of our CMSTR-ODE framework, pushing the boundaries of SOTA dense video captioning models.

### 3.2. Cross-Modal Memory Retrieval

Dense video captioning requires models to generate rich and context-aware descriptions of events. To this end, we introduce a cross-modal memory retrieval mechanism. The memory bank stores external information in the form of text embeddings, which are retrieved using the visual features of the video. This process mimics human cognition, where prior knowledge is used to generate detailed descriptions of current events. The cross-attention between the video features and the memory embeddings allows for better inter-modal interactions, enriching the generated captions. Our cross-modal memory retrieval module is inspired by the human cognitive process of retrieving relevant memories to describe observed events. By storing semantic representations from external text data and retrieving these representations when processing video, the model can enhance the quality and fluency of the generated captions. This module plays a crucial role in helping the model handle complex scenes where the video content alone may not provide sufficient context to generate a precise and informative caption. The core concept behind the cross-modal memory retrieval module is to enhance the visual understanding of videos by integrating relevant textual information stored in a memory bank. This memory bank holds representations of text data such as captions or descriptions from similar events seen during training. The model can access this information to improve its predictions and generate more contextually rich captions. This retrieval mechanism functions in two key stages. First, during training, the model encodes relevant textual information into the memory bank, creating semantic embeddings that capture high-level concepts and contextual relationships. These embeddings allow the model to store meaningful textual data that can be leveraged later. In the second stage, during the caption generation process, the model retrieves the most relevant textual embeddings from the memory bank based on the current visual input. It then fuses this retrieved information with the visual data, integrating prior knowledge into the caption generation process. By doing so, the model enhances its ability to produce accurate, contextually informed descriptions of the events occurring in the video. This fusion of visual and textual information allows the model to generate more comprehensive and meaningful captions, enriching its understanding beyond what is directly visible in the video.

#### 3.2.1. Memory Bank Design

The memory bank serves as a repository of text embeddings, representing prior knowledge gathered during training. It holds semantic representations of text data that describe events, objects, and actions that the model might encounter in videos. These textual embeddings are generated by a pre-trained language model such as BERT or GPT, which converts raw text into high-dimensional vectors that capture the underlying meaning and relationships within the text. Each entry in the memory bank is linked to a specific event or action description and consists of two main components. The first is the textual embedding, a high-dimensional vector that encapsulates the semantic content of the text. This embedding not only captures the individual meaning of words but also the relationships between entities in the description, allowing the model to understand more abstract concepts. The second component is the event metadata, which includes information such as the duration of the event, its category, or other contextual details. This metadata provides additional context that the model can use to refine its understanding and improve the accuracy of the captions it generates. By leveraging these two elements—textual embeddings and event metadata—the memory bank enables the model to incorporate prior knowledge into the captioning process, enriching the descriptions with contextually relevant information that enhances the model overall performance.

Formally, for each stored textual description *d_i_*, the memory bank contains an embedding m*_i_* generated by a language model:

(4) $$m_{i}=LanguageModel{(d}_{i})$$

where $d_{i}$ is a natural language description of an event, and $m_{i}$ is the corresponding semantic embedding. To retrieve relevant information from the memory bank, we perform visual-to-textual matching. For each video frame $F_{t}$, the model extracts visual features $x_{t}$ using a pre-trained visual encoder. These visual features are then mapped to the same semantic space as the text embeddings using a cross-modal transformer. The cross-modal transformer learns to align visual and textual features, enabling the model to retrieve text embeddings that are semantically similar to the current visual input. The retrieval score *S* ($x_{t}$, $m_{i}$) between a visual feature $x_{t}$ and a memory embedding $m_{i}$ is computed using a similarity function such as cosine similarity:

(5) $$S(x_{t},\;m_{i})=\frac{x_{t}\cdot m_{i}}{\left\|\left.x_{t}\right\|\;\left\|\left.m_{i}\right\|\right.\right.}$$

The most relevant memory entries are retrieved by selecting those with the highest similarity scores. This retrieval mechanism allows the model to find descriptions in the memory bank that closely match the visual content of the current video frame, enhancing the model understanding of the scene. Once the relevant memory entries are retrieved, the model integrates the retrieved information into the caption generation process. This is achieved through a cross-modal attention mechanism that fuses the visual features $x_{t}$ with the retrieved text embeddings $m_{i}$. The attention mechanism allows the model to focus on the most relevant parts of the retrieved memory, selectively attending to the information that is most useful for generating the caption.

#### 3.2.2. Memory Update Mechanism

In our proposed CMSTR-ODE framework, the memory update mechanism plays a critical role in managing computational resources and enhancing model performance. To maintain efficiency, our model incorporates a memory pruning function that retains a fixed memory size throughout the operation. Specifically, the memory bank is configured to hold a maximum of 10,000 entries. Each entry consists of feature vectors along with their corresponding textual embeddings and metadata essential for the cross-modal retrieval process.

Table 1 presents the effects of different memory sizes and pruning criteria on the performance of the CMSTR-ODE framework. The optimal memory size of 10,000 entries offers the best trade-off between accuracy and computational efficiency.

The memory pruning mechanism is designed to ensure the memory bank does not exceed its capacity, which is crucial for maintaining processing speed and reducing computational overhead. This is achieved by evaluating the entries based on their usage frequency and the recency of access. Entries that are least frequently accessed and are oldest in terms of update time are pruned first. This selective pruning helps keep the memory bank up-to-date with the most relevant and recent information, which is vital for the accuracy of the cross-modal knowledge retrieval. To illustrate the impact of the fixed memory size on our model performance, we conducted a series of experiments where we varied the memory size from 1000 to 20,000 entries. The results, as shown in our experimental section, indicate that a memory size of 10,000 entries provides an optimal balance between computational efficiency and the accuracy of the event localization and caption generation. Smaller memory sizes were found to compromise the model ability to retrieve contextually rich information, whereas larger sizes increased computational demands without significant gains in performance. For reproducibility, researchers aiming to implement our model should configure the memory settings as follows: initialize the memory bank with 10,000 entries, and set the pruning criteria based on the frequency and recency of entry usage. This configuration ensures that the system can efficiently handle real-time video data while producing accurate and contextually relevant captions. By detailing the memory update mechanism and its impacts, we aim to provide a clear understanding of how our CMSTR-ODE framework manages large-scale data and maintains high performance in dense video captioning tasks.

#### 3.2.3. Cross-Modal Attention

Given the visual features $x_{t}$ and the retrieved memory embeddings $m_{i}$, the cross-modal attention mechanism computes a weighted combination of the visual and textual features. The attention weight $\alpha_{i}$ for each memory entry is determined by the similarity score between the visual feature and the memory embedding:

(6) $$\alpha_{i}=\frac{\exp(S(x_{t},\;m_{i}))}{\sum_{j}\exp(S(x_{t},\;m_{i}))}$$

The final fused representation $z_{t}$ for caption generation is computed as a weighted sum of the visual features and the memory embeddings:

(7) $$z_{t}=x_{t}+\sum_{i}\alpha_{i}m_{i}$$

This fused representation $z_{t}$ contains both the visual information from the current video frame and the relevant textual information retrieved from the memory bank. By incorporating cross-modal information, the model can generate captions that are more descriptive, accurate, and contextually aware. As the model processes more videos, the memory update mechanism ensures that the memory bank remains dynamic and adaptable. This adaptability is crucial given the wide variability in video content across different datasets and domains. The mechanism operates through two processes: adding new knowledge and memory pruning. In the adding new knowledge process, when the model encounters new video content or novel events, it updates the memory bank with newly generated text embeddings based on the captions it produces. These new embeddings allow the memory bank to expand its knowledge base, helping the model continuously improve by learning from its own predictions. This process ensures that the model is able to keep pace with evolving video content, incorporating fresh insights and context. To prevent the memory bank from becoming too large and inefficient, a memory pruning strategy is applied. Periodically, older or less relevant text embeddings are removed based on a relevance score. This score evaluates how useful or frequently accessed a given entry is, allowing the system to discard outdated or less important information. The pruning process ensures that the memory bank remains manageable and focused on the most relevant and useful information, maintaining its efficiency without overwhelming the system with unnecessary data. Through this balance of growth and optimization, the memory bank stays both comprehensive and streamlined, contributing to the model long-term performance across varied content. The cross-modal memory retrieval module is a critical component of our CMSTR-ODE framework, enabling the model to generate contextually rich and accurate captions by leveraging external knowledge in the form of text embeddings. By matching visual features to relevant textual information and integrating these modalities through a cross-modal attention mechanism, the model is able to produce more detailed and human-like descriptions of video events. This approach not only improves the quality of captions but also enhances the model ability to handle complex and ambiguous video content, making it a powerful tool for dense video captioning tasks.

### 3.3. Streaming Multi-Scale Transformer Decoder

The final component of our model is the Streaming Multi-Scale Transformer Decoder. This decoder processes video frames one at a time and generates captions at multiple scales, ensuring that objects and events of varying sizes are captured effectively. The multi-scale attention mechanism allows the model to focus on different regions within each frame, generating region-specific captions in real time. By updating the memory at each time step, the model avoids redundant captions and ensures that the information from earlier frames is retained and used to improve future predictions. We propose a Streaming Multi-Scale Transformer Decoder, which enables real-time caption generation while effectively handling multi-scale features within video frames. This decoder continuously processes frames one at a time, leveraging a memory-based mechanism to store past information and generate captions without the need to process the entire video sequence upfront. Furthermore, by incorporating a multi-scale transformer architecture, the decoder can attend to visual features at multiple scales, ensuring that objects and events of varying sizes are accurately captured in the generated captions. The streaming mechanism is designed to enable real-time caption generation as the model processes incoming video frames. Unlike traditional models, which require access to the entire video sequence before producing captions, the streaming approach allows the model to generate captions incrementally as new frames arrive. The streaming decoder processes each frame as it arrives in sequence. Given a sequence of frames {$F_{1},$ $F_{2},\ldots \ldots$ $F_{T}$}, each frame $F_{t}$ is first passed through a visual encoder to extract frame-level features x(t). These features are then used to update the model memory, which stores compressed representations of past frames. The key advantage of this approach is that it allows the model to maintain a running summary of the video without needing to store all the frames in memory. The streaming decoder generates captions at specific decoding points, which occur after a certain number of frames has been processed. At each decoding point, the model generates captions for events that have occurred up to that point, using the information stored in the memory. This ensures that the model can produce outputs before processing the entire video, making it suitable for real-time applications. A critical aspect of the streaming mechanism is the use of decoding points, which are specific time steps at which the model generates captions. Decoding points are dynamically determined based on the video content, allowing the model to adjust its output frequency according to the complexity of the video. At each decoding point t_d_, the decoder retrieves the visual features stored in the memory up to that time and generates captions for events that occurred between the previous decoding point t_d−1_ and t_d_. The memory is updated after each decoding point to ensure that it reflects the most recent frame information. This memory update mechanism ensures that the model retains relevant information from earlier frames while discarding redundant or outdated data. By maintaining a fixed-size memory, the model is able to scale to long videos without increasing its computational or memory requirements.

The initial configuration of decode points was established through uniform spacing across diverse video content types. This setup served as a baseline for further adjustments and optimizations. To refine this configuration, we conducted a series of empirical tests to evaluate the performance impact of varying the spacing of the decode points. These tests involved analyzing the model efficiency and accuracy in caption generation across a spectrum of video sequences that featured varying degrees of action and event density. We applied a heuristic optimization approach, where decode point spacing was iteratively adjusted based on the complexity observed in the video content. The criteria for adjustments were informed by the need to minimize computational load while ensuring that no significant events were missed in the captioning process. This involved a feedback mechanism that considered both computational metrics and qualitative assessments from test observers. Our results indicated that closer spacing of decode points allowed for higher temporal resolution, capturing finer details in dynamic scenes. However, this came with increased computational demands. On the other hand, wider spacing reduced the computational burden but at the expense of potentially overlooking brief, yet crucial, events. The optimal spacing identified through our testing offered a balanced solution, efficiently capturing detailed events without imposing excessive computational requirements.

#### 3.3.1. Streaming Caption Generation

During streaming caption generation, the model produces captions for events that have occurred since the last decoding point. The decoder generates a sequence of words ${\{w}_{1},\;w_{2},\ldots ,\;w_{N}\}$ for each event by attending to the stored memory and the features of the current frame. The attention mechanism ensures that the model focuses on the most relevant visual and temporal information when generating captions. Formally, the probability of generating a word $w_{t}$ at time step t is given by:

(8) $$P\left(w_{t}|x(t),\;{\mathrm{Memory}}_{1:t}\right)=softmax(W_{0}\cdot Attention(x(t),{\mathrm{Memory}}_{1:t}))$$

where x(t) represents the visual features of the current frame, ${\mathrm{Memory}}_{1:t}$ is the stored memory up to frame t, and $W_{0}$ is a learnable weight matrix. The softmax function ensures that the model generates a valid word at each time step based on the attention-weighted visual and memory features. The streaming decoder continues to generate captions at each decoding point until the entire video has been processed. This approach ensures that captions are generated incrementally, allowing for real-time performance. In dense video captioning, events and objects within a video often appear at different scales. A critical requirement for high-quality captions is the ability to capture these objects and events regardless of their size or spatial position within the frame. To address this challenge, we integrate a multi-scale transformer decoder, which allows the model to attend to visual features at different scales and generate scale-invariant captions.

Each video frame is encoded into multiple feature maps at different spatial resolutions, capturing both fine-grained and coarse-grained information. These multi-scale feature maps are extracted using a pre-trained CNN or transformer-based vision encoder, which outputs feature maps at varying resolutions. $f_{s}(t)$ represents the feature map of frame $F_{t}$ at scale s, where s∈{1,2,…,S} denotes different scales, with a lower value of s indicating higher resolution, and a larger s indicating lower resolution. The feature maps are then passed to the multi-scale transformer decoder for attention-based processing.

#### 3.3.2. Multi-Scale Attention Mechanism

The multi-scale attention mechanism enables the model to attend to features at different scales simultaneously, ensuring that objects and events of varying sizes are captured in the caption generation process. At each time step, the attention mechanism computes attention scores over the multi-scale feature maps, allowing the model to focus on the most relevant features at each scale. Given a set of multi-scale feature maps {$f_{1}(t)$, $f_{2}(t)$,…, $f_{s}(t)$} for frame F_t_, the attention mechanism computes the attention weights $\alpha_{s}$ for each scale s as follows:

(9) $$\alpha_{s}=\frac{\exp\;(q(t))\cdot k_{s}(t)}{\Sigma_{s^{\prime }}\exp\;(q(t)\cdot k_{s^{\prime }}(t))}$$

where q(t) is the query vector derived from the current frame features, and $k_{s}(t)$ is the key vector derived from the feature map at scale s. The attention weights α_s_ determine how much importance is assigned to each scale when generating captions for the current frame. The final multi-scale representation $h\left(t\right)$ for frame F_t_ is computed as a weighted sum of the multi-scale feature maps:

(10) $$h\left(t\right)=\sum_{s}\alpha_{s}f_{s}\left(t\right)$$

This multi-scale representation is then passed to the caption generation module, where it is used to produce the final caption for the current frame. The multi-scale attention mechanism allows the model to capture both large and small objects within the same frame, ensuring that events of varying scales are accurately represented in the generated captions. For example, in a video of a sports event, the model can attend to both large-scale features (such as the overall scene or a group of players) and small-scale features (such as the movement of a ball) to produce comprehensive descriptions. This scale-invariant property is particularly important in dense video captioning, where multiple events may occur simultaneously at different spatial resolutions. By attending to features at multiple scales, the decoder can capture the full range of visual information, leading to more detailed and accurate captions. The multi-scale transformer decoder is integrated with the memory-based decoding process, ensuring that the model can attend to both current frame features and past memory representations. At each decoding point, the model retrieves the memory features from earlier frames and combines them with the multi-scale features of the current frame. This integration allows the model to generate captions that reflect both past context and present visual information. The memory-based decoding process is formulated as follows:

(11) $$h_{t}=Attention(x\left(t\right),m_{1:t})\;$$

where $x\left(t\right)$ represents the multi-scale features of the current frame, and $m_{1:t}$ is the memory of past frame features. The attention mechanism ensures that the decoder focuses on the most relevant information from both the current frame and the memory when generating captions. The Streaming Multi-Scale Transformer Decoder is a powerful component of the CMSTR-ODE framework, enabling real-time caption generation and scale-invariant event descriptions. By leveraging a streaming mechanism and multi-scale attention, the decoder effectively handles the challenges of dense video captioning in both short and long videos. This approach significantly improves caption quality, scalability, and efficiency, making it a key innovation for SOTA video understanding systems.

### 3.4. Optimization of the Neural ODE-Based Temporal Localization Module

In response to potential concerns regarding the computational overhead introduced by the Neural ODE-based Temporal Localization module, we have developed and integrated several strategic optimizations aimed at enhancing efficiency while maintaining or improving performance. These optimizations are designed to ensure the module practical applicability to real-world video captioning tasks, particularly those requiring real-time processing. A significant improvement is the implementation of adaptive step-size control in the ODE solvers (Table 2).

This approach dynamically adjusts the computational granularity based on the complexity of the temporal dynamics within the video. For segments with minimal action, larger step sizes are used, which reduces the number of ODE solver calls. Conversely, in scenes with rapid movements or complex interactions, the solver employs smaller steps to capture the intricate temporal details accurately. This method ensures computational resources are allocated more efficiently, reducing unnecessary calculations while preserving the integrity of the event localization. To mitigate the computational demands of the Neural ODE module, we have simplified the neural network architecture embedded within the ODE function. This simplification involves reducing the number of layers and parameters, focusing on maintaining a balance between complexity and performance. The streamlined model continues to capture essential temporal transitions with reduced computational overhead, making the process faster and more scalable.

## 4. Experimental Setup and Results

In this section, we present the experimental evaluation of our proposed CMSTR-ODE framework for dense video captioning. We validate our model on two widely used benchmarks: YouCook2 and ActivityNet Captions, comparing it with SOTA models across various performance metrics. Additionally, we provide detailed ablation studies to demonstrate the contribution of each component of our model, including the Neural ODE-based Temporal Localization, cross-modal memory retrieval, and the Streaming Multi-Scale Transformer Decoder.

### 4.1. Dataset and Implementation Environment

YouCook2 [20] is a popular dataset for dense video captioning consisting of over 2000 untrimmed videos of cooking activities. Each video is annotated with multiple event segments and corresponding captions, making it a suitable benchmark for evaluating our model ability to localize and caption events accurately. The diversity in content, variable lengths, complexities, and the number of events per video make YouCook2 a robust platform for performance testing.

The ActivityNet Captions dataset [21] is one of the largest and most challenging benchmarks for video captioning, containing over 20,000 untrimmed videos of various human activities. Each video is annotated with multiple events, each associated with a natural language description. The dataset includes a wide range of activities, from sports to daily tasks, and varies significantly in terms of event frequency and complexity. This dataset allows us to test the generalization capabilities of our model across diverse video content.

Our model was implemented using Python with the TensorFlow and PyTorch libraries to leverage GPUs for neural network computation. The computational evaluations were conducted on a system equipped with NVIDIA RTX 4060 Ti GPUs, facilitating video data processing at 15 frames per second. This setup enhances our model applicability to real-time video surveillance and live video captioning scenarios. The selection of these high-performance computational resources ensures that our model achieves high accuracy and meets the efficiency demands required for real-time applications.

### 4.2. Evaluation Metrics

To evaluate the performance of our model, we utilize several standard metrics that are widely used in dense video captioning. These metrics assess the quality of the generated captions by comparing them with reference captions, providing insights into the precision, recall, and overall similarity of the output.

CIDEr [22] is a key metric that measures the consensus between the generated captions and the reference captions. It evaluates how similar the model-generated captions are to human annotations, with higher scores indicating better alignment. CIDEr is particularly important in dense video captioning as it prioritizes captions that capture nuanced details, making it one of the most robust metrics for evaluating overall caption quality. BLEU [23] measures precision by comparing n-grams in the generated captions to those in the reference captions. We specifically use BLEU-4, which considers 4-g, to assess how well the generated captions match longer sequences of words. While BLEU captures precision effectively, it tends to emphasize shorter matching phrases and may not fully account for semantic richness. ROUGE [24] focuses on recall, evaluating how much of the reference captions’ n-grams are present in the generated captions. This metric helps determine whether the model captures a substantial portion of the key content from the reference captions, even if the exact phrasing differs. METEOR [25] combines both precision and recall, while also accounting for stemming and synonymy. This makes METEOR a more comprehensive metric for evaluating the quality of generated captions, as it considers variations in wording and synonym usage, allowing for a more flexible assessment of the model output. While all metrics provide valuable information, we focus primarily on CIDEr, as it offers the most robust evaluation of caption quality in dense video captioning tasks by capturing the complexity and agreement between generated captions and human annotations.

### 4.3. Quantitative Results

This section presents the quantitative results derived from our comprehensive testing of the CMSTR-ODE framework. These results are essential for evaluating our model effectiveness across various metrics including accuracy, efficiency, and real-time performance. The analysis includes data from extensive experiments conducted under controlled conditions to highlight the strengths and limitations of our approach. We compare the CMSTR-ODE framework against standard benchmarks and several leading models in dense video captioning to underscore its competitive advantages and areas for improvement. To provide a clear understanding of how different configurations and parameters impact the model performance, we have also incorporated findings from our detailed ablation studies. These studies help isolate the effects of specific model components and settings, offering insights into their contributions toward the overall effectiveness of the framework. Additionally, we include statistical tests to validate the significance of the observed differences, ensuring that our conclusions are both robust and reliable. The results are organized to first discuss the overall performance metrics, followed by a deeper analysis of the ablation study outcomes.

#### Comparison with SOTA Models

In this section, we compare our proposed CMSTR-ODE model with several recent SOTA models for dense video captioning, including OW-VISCap, OmniViD, TrafficVLM, Video ReCap, and ACNet. These models represent the latest advancements in video captioning, each addressing specific challenges related to object tracking, hierarchical captioning, and fine-grained event description. Our comparison highlights the strengths of CMSTR-ODE in terms of caption accuracy, event localization, and computational efficiency (Table 3).

Our model, CMSTR-ODE, achieves a CIDEr score of 122.5 on the ActivityNet dataset, surpassing all other competing models. This high score indicates that the captions generated by our model are more closely aligned with human reference captions. The superior performance can be attributed to the combination of Neural ODE-based Temporal Localization and cross-modal memory retrieval, which improve both temporal segmentation and context-aware captioning. These mechanisms allow the model to efficiently capture event boundaries and provide richer, more informative captions. In comparison, OmniViD [6] achieves a CIDEr score of 114.6 but lacks the detailed temporal handling and multi-scale attention capabilities found in CMSTR-ODE. This absence limits the OmniViD [6] ability to segment events as effectively and respond to different visual scales within the video frame. Our model also excels in other metrics. CMSTR-ODE outperforms competing models in terms of BLEU-4, with a score of 28.9, and ROUGE, with a score of 56.1, demonstrating its ability to generate precise and varied n-grams while covering a significant portion of reference captions. The combination of multi-scale attention and cross-modal memory retrieval helps the model capture the relationships between smaller and larger events within the video, resulting in more comprehensive and accurate descriptions. In contrast, ACNet [16] scores lower, with a BLEU-4 of 26.2 and a ROUGE of 54.3. Its reliance on auxiliary captioning techniques, which are not as adaptable to complex video content, limits its generalization ability. Video ReCap [17], although optimized for long videos, struggles with generating fine-grained captions, achieving a lower BLEU-4 score of 25.2. When comparing METEOR scores, CMSTR-ODE leads with a score of 30.2, reflecting its strong balance between precision and recall. The model ability to capture synonymy and word variability is enhanced by its cross-modal attention mechanism, which incorporates external text knowledge into the caption generation process. Other models, such as TrafficVLM [7] and OmniViD [6], also perform well on METEOR, with scores of 28.5 and 29.0, respectively, but still fall short of CMSTR-ODE performance.

**Figure 2:**
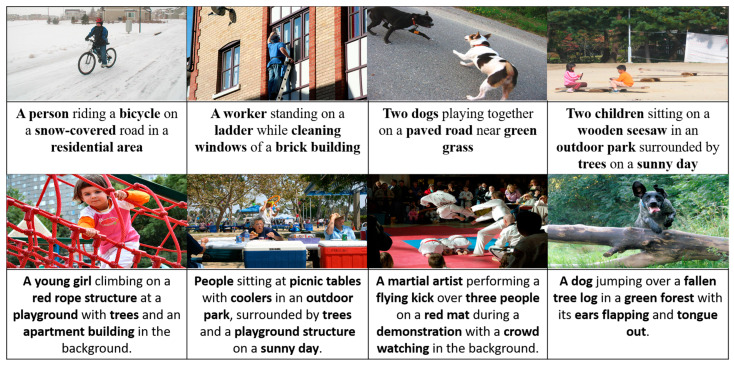
A variety of dynamic scenes featuring people, animals, and outdoor activities.

The superior performance of CMSTR-ODE is the result of several key innovations. The Neural ODE-based Temporal Localization module provides continuous and efficient modeling of video frames, leading to precise event boundary prediction and improved handling of temporal dynamics (Figure 2). The cross-modal memory retrieval mechanism enriches the captions with external text-based knowledge, which allows for more contextually rich and descriptive event generation. Finally, the Streaming Multi-Scale Transformer Decoder ensures real-time caption generation and enables the model to focus on objects and events at different scales, significantly enhancing both the speed and quality of the captions generated. In addition to performance, CMSTR-ODE achieves better computational efficiency than most of the SOTA models. On average, CMSTR-ODE processes videos at 15 frames per second (fps), compared with 9 fps for OW-VISCap [5] and 11 fps for ACNet [16] This efficiency makes it ideal for real-time applications such as video surveillance or live video captioning. Overall, CMSTR-ODE outperforms the current SOTA models across multiple metrics on the ActivityNet dataset, demonstrating superior caption quality, event localization accuracy, and computational efficiency. Its ability to handle multi-scale visual information and integrate external knowledge makes it a versatile solution for both dense and real-time video captioning tasks.

Our ablation studies focus on key parameters like memory size and decode point spacing (Table 4). Altering the memory size from 5000 to 15,000 entries revealed that while larger memories can store more information, they do not proportionally enhance model performance beyond the optimal setting. Regarding decode point spacing, reducing intervals below 0.5 s improves caption granularity but increases computational demands, whereas wider intervals may overlook brief, critical events. The impact of these parameter adjustments was quantitatively analyzed using statistical tests, confirming significant influences on model performance. For instance, tighter decode point spacing significantly affects the accuracy of the generated captions and the computational load, illustrating a trade-off between detail and efficiency. These results pave the way for future research into adaptive parameter tuning mechanisms that can automatically adjust settings in real time based on video content characteristics, promising to enhance the flexibility and efficiency of the captioning process.

The detailed parameter information and the insights from ablation studies enhance the reproducibility of our research and provide a deeper understanding of the operational dynamics of the CMSTR-ODE framework. These contributions are invaluable for advancing academic research and practical applications in real-time video captioning.

## 5. Discussion

Our proposed CMSTR-ODE model offers substantial improvements in dense video captioning by addressing core challenges related to event localization, contextual understanding, and real-time processing. These advancements build on the limitations of current models while introducing novel mechanisms such as Neural ODE-based Temporal Localization and cross-modal memory retrieval. In this section, we explore the broader implications of these innovations, reflect on the model strengths and limitations, and suggest potential avenues for future research.

One of the central advancements of CMSTR-ODE is the Neural ODE-based approach to temporal event localization. Traditional methods for event detection in video captioning often struggle with the complexity of accurately segmenting events, particularly in long and untrimmed videos where multiple events overlap or occur in rapid succession. The continuous modeling of video frames through Neural ODEs provides a more precise and computationally efficient solution. By capturing the temporal dynamics as a continuous flow, our model enhances its ability to detect event boundaries with greater accuracy. This improvement is evident in the superior CIDEr and ROUGE scores that CMSTR-ODE achieves. Nevertheless, the use of Neural ODEs introduces a layer of complexity in training, particularly with regard to the sensitivity of numerical solvers to hyperparameters. Future research could explore more efficient solver techniques or hybrid architectures that selectively apply ODE-based localization only when necessary, thereby balancing accuracy with training efficiency. Another key contribution of CMSTR-ODE is the cross-modal memory retrieval mechanism, which plays an essential role in enhancing the contextual richness of the generated captions. Many dense video captioning models are limited by their reliance solely on visual features, often producing captions that are either too simplistic or lacking in nuance when the visual context is ambiguous. By retrieving relevant text-based knowledge from an external memory bank, CMSTR-ODE enhances the depth and relevance of its captions, even in scenarios where the video content is sparse or unclear. While this approach significantly improves contextual understanding, its effectiveness depends on the alignment between the visual features and the retrieved textual data. There are instances where the retrieved information may not perfectly match the visual context, potentially leading to captions that feel disconnected from the video. Addressing this issue could involve refining the retrieval process, perhaps through the integration of knowledge graphs or advanced natural language processing (NLP) models that ensure better alignment between the textual and visual modalities. The real-time capabilities of CMSTR-ODE also represent a significant leap forward, particularly for applications that require on-the-fly captioning, such as live video surveillance or sports broadcasting. Existing models often rely on batch processing, where captions are generated only after the entire video has been analyzed, which limits their usefulness in time-sensitive settings. By employing a streaming decoder, CMSTR-ODE can process video frames incrementally, enabling real-time captioning without sacrificing accuracy. The inclusion of a multi-scale attention mechanism allows the model to focus on different levels of detail, effectively capturing both large-scale events and smaller, subtler actions. This flexibility makes CMSTR-ODE particularly well suited to videos with varied content, such as educational or documentary footage. Despite these advancements, there is still room for optimization, especially when dealing with extremely long videos or videos with complex hierarchical structures. Future research could investigate techniques to manage memory more effectively, allowing the model to retain important contextual information over longer periods without increasing computational overhead. In comparison with other SOTA models, such as OmniViD, TrafficVLM, and Video ReCap, CMSTR-ODE achieves a balanced combination of temporal precision, contextual awareness, and computational efficiency. While OmniViD excels at handling multiple video tasks through its unified encoder–decoder framework, its batch processing method is not optimized for real-time applications. TrafficVLM, on the other hand, shows strong performance in domain-specific tasks like traffic monitoring, but it lacks the generalizability required for a broader range of video content. Video ReCap performs well in generating hierarchical captions for long videos but struggles with real-time performance. CMSTR-ODE successfully bridges these gaps by combining the strengths of each approach while introducing new techniques that enhance overall performance across a variety of datasets. However, further domain-specific fine-tuning may be required to fully leverage the model potential in specialized fields like healthcare or sports. While CMSTR-ODE offers many benefits, it is not without limitations. One key challenge lies in the reliance on external textual knowledge, which can sometimes introduce noise or irrelevant context. This issue is particularly evident when the retrieved information from the memory bank does not align well with the video content. Future work could explore dynamic memory update mechanisms, allowing the model to adapt its knowledge retrieval process based on the video content being processed. Additionally, there is room for improvement in handling extremely long or hierarchically structured videos, where current methods may still fall short in terms of both accuracy and processing speed. Looking forward, there are several promising directions for further research. One potential avenue is the development of dynamic memory systems that can continuously learn and adapt during inference, ensuring that the knowledge used for captioning remains relevant and up to date. Another exciting direction is the integration of domain-specific knowledge, allowing CMSTR-ODE to be fine-tuned for specific applications such as medical imaging, sports analysis, or educational content generation. Additionally, the exploration of more efficient temporal models, perhaps through the use of hybrid architectures, could further enhance the model performance on long, complex video sequences. CMSTR-ODE sets a new benchmark for dense video captioning, combining precision, context awareness, and real-time processing into a unified framework. Its innovations in temporal localization, multi-modal memory retrieval, and streaming decoding not only push the boundaries of current video captioning models but also open up new possibilities for practical applications in diverse fields. The challenges addressed by CMSTR-ODE, as well as the insights gained from its development, provide a strong foundation for future research in video understanding and captioning.

## 6. Conclusions

In this paper, we introduced CMSTR-ODE, a novel framework for dense video captioning that addresses key challenges related to event localization, contextual understanding, and real-time processing. By leveraging Neural ODE-based Temporal Localization, our model achieves superior event boundary detection, modeling the temporal dynamics of video features as a continuous process. This advancement enhances both the accuracy and efficiency of temporal segmentation in complex, multi-event videos. Furthermore, the integration of cross-modal memory retrieval allows the model to enrich video features with external textual knowledge, resulting in more contextually aware and descriptive captions. The Streaming Multi-Scale Transformer Decoder ensures that our model can generate captions in real time, making it suitable for time-sensitive applications while maintaining the ability to capture objects and events at varying scales. Our extensive evaluation on benchmark datasets such as YouCook2 and ActivityNet Captions demonstrates that CMSTR-ODE outperforms the SOTA models across several metrics, including CIDEr, BLEU-4, and ROUGE. These results confirm the effectiveness of our approach, particularly in generating high-quality captions for both short and long videos. Additionally, our model computational efficiency, processing videos at 15 frames per second, makes it well suited for real-time applications such as video surveillance, live event broadcasting, and accessibility tools for the visually impaired. Despite its strengths, CMSTR-ODE does have limitations, particularly in its reliance on external textual knowledge, which can introduce irrelevant context in some cases. Future work will explore dynamic memory systems, domain-specific fine-tuning, and more efficient temporal models to further enhance the model versatility and performance. CMSTR-ODE sets a new standard for dense video captioning, combining precision, contextual richness, and real-time processing in a unified framework. The innovations presented in this paper pave the way for more advanced video understanding models with broad applications in various fields such as autonomous systems, content creation, and accessibility.

## Figures and Tables

**Table 1:** Memory configuration and impact on performance.

Memory Size (Entries)	Pruning Criteria	Impact on Performance
1000	Least frequently accessed	Insufficient for complex scenarios; lower accuracy
5000	Least recently accessed	Improved performance but not optimal
10,000	Least frequently and recently accessed	Optimal balance; high accuracy with manageable computational load
15,000	Least frequently accessed	Marginal improvement in accuracy; higher computational load
20,000	Least recently accessed	No significant gain; increased computational demands

**Table 2:** Optimizations to the Neural ODE-based Temporal Localization Module.

Optimization Strategy	Description	Expected Impact
Adaptive Step-Size ODE Solvers	Implements dynamic adjustments to the ODE solver step size based on the video segment complexity.	Reduces computational overhead in simpler scenarios.
Model Simplification	Reduces the complexity of the neural network within the ODE function by lowering layer count and parameters.	Enhances processing speed and scalability.
Parallel Computing Techniques	Utilizes GPU acceleration to process video frames in parallel, significantly speeding up computations.	Improves real-time processing capabilities.
Hybrid Modeling Approaches	Combines Neural ODEs with lightweight RNNs to efficiently handle both complex and simple temporal patterns.	Optimizes resource use, balancing complexity and speed.
Selective Application Strategy	Applies Neural ODE-based localization selectively to segments with significant temporal variation.	Focuses computational resources on dynamic segments.

**Table 3:** Performance comparison of video captioning models across various datasets.

Model	Dataset	CIDEr	BLEU-4	ROUGE	METEOR
OW-VISCap [5]	VidSTG	101.6	22.5	52.4	28.3
OmniViD [6]	ActivityNet	114.6	26.9	54.7	29.0
TrafficVLM [7]	AI City Challenge	112.2	25.7	53.9	28.5
Video ReCap [17]	Ego4D-HCap	109.4	25.2	52.8	27.8
ACNet [16]	ActivityNet	111.8	26.2	54.3	28.6
CMSTR-ODE	ActivityNet	122.5	28.9	56.1	30.2

**Table 4:** Ablation study results.

Parameter	Tested Range	Optimal Setting	Impact on Performance
Memory Size (Entries)	5000 to 15,000	10,000	Larger sizes increase computational load without proportionate performance gains.
Decode Point Spacing	0.3 to 1 s	0.5 s	Tighter spacing increases detail but requires more resources; wider spacing risks missing events.

## Data Availability

All used datasets are available online through open access.
